# LRP11 facilitates lipid metabolism and malignancy in hepatocellular carcinoma by stabilizing RACK1 through USP5 regulation

**DOI:** 10.1186/s10020-025-01097-6

**Published:** 2025-01-31

**Authors:** Litao Liang, Wenbo Jia, Jinyi Wang, Yanzhi Feng, Deming Zhu, Wenhu Zhao, Chao Xu, Xiangyu Ling, Qingpeng Lv, Xiaoming Ai, Lianbao Kong, Wenzhou Ding

**Affiliations:** 1 Hepatobiliary Center, The First Affiliated Hospital of Nanjing Medical University, 300 Guangzhou Road, Nanjing, Jiangsu China; 2 Key Laboratory of Liver Transplantation, Key Laboratory of Hepatobiliary Cancers, Chinese Academy of Medical Sciences, National Health Commission (NHC, Nanjing, Jiangsu China; 3 Department of Hepatobiliary Pancreatic Spleen Surgery, Affiliated Hospital of Jiangsu University, No.438 Jiefang Road, Zhenjiang, Jiangsu China

**Keywords:** LRP11, Hepatocellular carcinoma, Deubiquitination, Lipid metabolism

## Abstract

Hepatocellular carcinoma (HCC) is one of the most prevalent cancers worldwide and a major public health challenge. Lipid metabolism plays a crucial role in the metabolic reprogramming observed in HCC, although the underlying mechanisms are still being elucidated. Nile red staining and lipid assays showed that LRP11 knockdown significantly reduces lipid accumulation in HCC cells, with a concurrent decrease in key lipid metabolism markers such as FSAN, ACLY and ACSL4, as demonstrated by Western blotting. Mass spectrometry (MS) and co-immunoprecipitation (Co-IP) revealed that LRP11 recruits USP5, enhancing USP5-mediated deubiquitination of RACK1. Truncation analysis identified LRP11 residues 309–500 as critical for interaction with the RACK1 residues 91–231. These findings suggest that LRP11 may influence lipid metabolism and progression in HCC through USP5-mediated stabilization of RACK1. Based on these results, LRP11 emerges as a potential target for further exploration in HCC therapy. Targeting LRP11 or disrupting its interactions with USP5 or RACK1 could offer new avenues for treatment, though additional research is required to validate these therapeutic possibilities.

**Supplementary Information:**

The online version contains supplementary material available at 10.1186/s10020-025-01097-6.

## Introduction

Primary hepatocellular carcinoma (HCC) is one of the six most common cancers globally and ranks third in cancer-related mortality, making it a significant global healthcare challenge (Llovet et al. 2021; Sung et al. 2021) Despite advancements in comprehensive treatment strategies, including surgical resection, ablation, chemoembolization, liver transplantation, and systemic therapies, the prognosis for HCC patients remains poor (Llovet et al. 2021). The asymptomatic nature of early-stage HCC and the lack of effective diagnostic methods often result in late-stage diagnosis, contributing to high mortality rates and poor patient prognosis. Therefore, understanding the molecular mechanisms underlying the development and progression of HCC is essential for identifying potential therapeutic targets, which may contribute to improving early diagnosis and patient outcomes in the future.

Our study focuses on low-density lipoprotein receptor-related protein 11 (LRP11), a member of the low-density lipoprotein receptor (LDLR) family (Roslan et al. 2019). The LDLR family consists of transmembrane proteins encoding single-pass receptors, and previous studies have shown that LDLR family genes are involved in various cellular processes and tumor progression (He et al. 2023; Lin et al. 2024; Liu et al. 2021, 2024; Rong et al. 2022). However, studies on LRP11 in tumorigenesis are limited, with evidence suggesting its involvement in the malignant progression of prostate and cervical cancers (Gan et al. 2020; Gu et al. 2023). To date, the mechanisms by which LRP11 contributes to HCC remain unreported, making it a critical area for further investigation in understanding HCC progression.

Receptor for activated C kinase 1 (RACK1) is a member of the tryptophan-aspartate repeat (WDR) protein family (Schapira et al. 2017). Previous studies have shown that RACK1 is a multifunctional scaffold protein that interacts with various receptor proteins and protein kinases, thereby regulating key cellular activities such as signal transduction, cell adhesion, and migration, in both physiological and pathological conditions (Li and Xie 2015; Cao et al. 2019; Duff and Long 2017). Dysregulation of RACK1 expression influences tumorigenesis, potentially promoting proliferation, metastasis, invasion, and chemoresistance in various cancers, including ovarian, breast, lung, osteosarcoma, hepatocellular, cervical, and oral squamous cell carcinoma (Pi et al. 2023; Tian et al. 2023; Wang et al. 2022; Xia et al. 2023; Peng et al. 2024; Wu et al. 2020; Dan et al. 2020). Furthermore, evidence suggests that RACK1 regulation is closely associated with the ubiquitin–proteasome system (Peng et al. 2024; Yu et al. 2021; Ou et al. 2022; Li et al. 2023). Zhouxia Ke et al. demonstrated that TRIM26 interacts with RACK1 and promotes its degradation through ubiquitination (Xia et al. 2023). Additionally, SMURF2 adds K6, K33, and K48 ubiquitin chains to RACK1, leading to its ubiquitination and instability (Pi et al. 2023). Nevertheless, the exact mechanisms by which RACK1 regulates the malignant progression of HCC remain unclear, highlighting the need for further investigation to explore its potential role in HCC.

In this study, we show that LRP11 is upregulated in HCC and is correlated with poor prognosis. Our data suggest that LRP11 may promote HCC cell proliferation and migration in both in vitro and in vivo models. Mechanistically, LRP11 appears to interact with RACK1, potentially facilitating its deubiquitination via USP5, thereby stabilizing RACK1 protein levels. This interaction may contribute to enhanced HCC proliferation and invasion. These findings propose a novel mechanism by which LRP11 may influence HCC progression, and targeting the LRP11-RACK1 axis could provide a potential avenue for further exploration in HCC treatment.

## Materials and methods

### Bioinformatics analysis

Gene expression levels, prognostic significance, and pathological grading in HCC and normal tissues were analyzed using HCC sample data from The Cancer Genome Atlas (TCGA) via the GEPIA (gepia.cancer-pku.cn) and UALCAN (ualcan.path.uab.edu) databases.

### Hepatocellular carcinoma tissue samples

The HCC and adjacent non-tumor tissues used in this study were obtained from the Hepatobiliary Center of the First Affiliated Hospital of Nanjing Medical University. All tissue samples were collected with written informed consent from the patients and were approved by the Ethics Committee of the First Affiliated Hospital of Nanjing Medical University.

### RNA sequencing

Total RNA was extracted from Hep3B cells in the negative control and LRP11-OE groups using TRIzol reagent (Invitrogen, California, USA). The purity and concentration of total RNA were measured using a NanoDrop 2000 spectrophotometer (Thermo Scientific, MA, USA). RNA-seq analysis was conducted by Hongxu Biotechnology Company (Shanghai, China).

### IP combined with mass spectrometry

Total protein was extracted from Hep3B cells, and immunoprecipitation (IP) was performed using an anti-LRP11 antibody (sc-514698, Santa Cruz) and protein A/G agarose beads (Thermo Scientific, MA, USA) as described above. Mass spectrometry analysis was conducted by BGI Tech Solutions Co., Ltd. (BGI, Shenzhen, Guangdong, China).

### Co-immunoprecipitation (Co-IP) assay

The collected Hep3B cells and HEK293T cells transfected with His-LRP11, Flag-RACK1, and HA-USP5 were resuspended in 1 mL of RIPA protein lysis buffer containing 1% protease inhibitor and incubated on ice for a minimum of 30 min. The supernatants were then harvested and incubated with the respective primary antibodies or IgG at 4 °C for 24 h. Protein A and G agarose beads (Thermo Scientific, MA, USA) were subsequently added, and the mixture was incubated at 4 °C for an additional 3 h. The beads were washed five times with PBS buffer, and 40 μL of sample loading buffer was added followed by boiling for 5 min. Western blot analysis was performed on the processed samples. The following antibodies were utilized: LRP11 (sc-514698, Santa Cruz, 1:200 dilution), USP5 (10473-1-AP, Proteintech, 1:300 dilution), RACK1 (27592-1-AP, Proteintech, 1:250 dilution), His-tag (66005-1-Ig, Proteintech, 1:200 dilution), Flag-tag (20543-1-AP, Proteintech, 1:200 dilution), and HA-tag (51064-2-AP, Proteintech, 1:200 dilution).

### Data analysis

Statistical analyses were performed using SPSS (version 19.0; SPSS, New York, USA) and GraphPad Prism 8.0 (GraphPad Software, California, USA). A two-tailed Student’s t-test was used to assess differences between two groups. Fisher's exact test was employed to determine the association between LRP11 expression levels and clinicopathological parameters in HCC patients. Spearman’s correlation analysis was used to evaluate the correlation between LRP11 and MAZ expression. All experiments were repeated at least three times independently, and data are presented as mean ± standard deviation. Statistical significance was set at p < 0.05 (n.s. = not significant, *p < 0.05, **p < 0.01, ***p < 0.001). All data collected were included in the final statistical analysis.

Additional materials and methods are provided in the Supplementary materials and methods.

## Results

### LRP11 upregulation is associated with HCC progression and poor prognosis

**Fig. 1:**
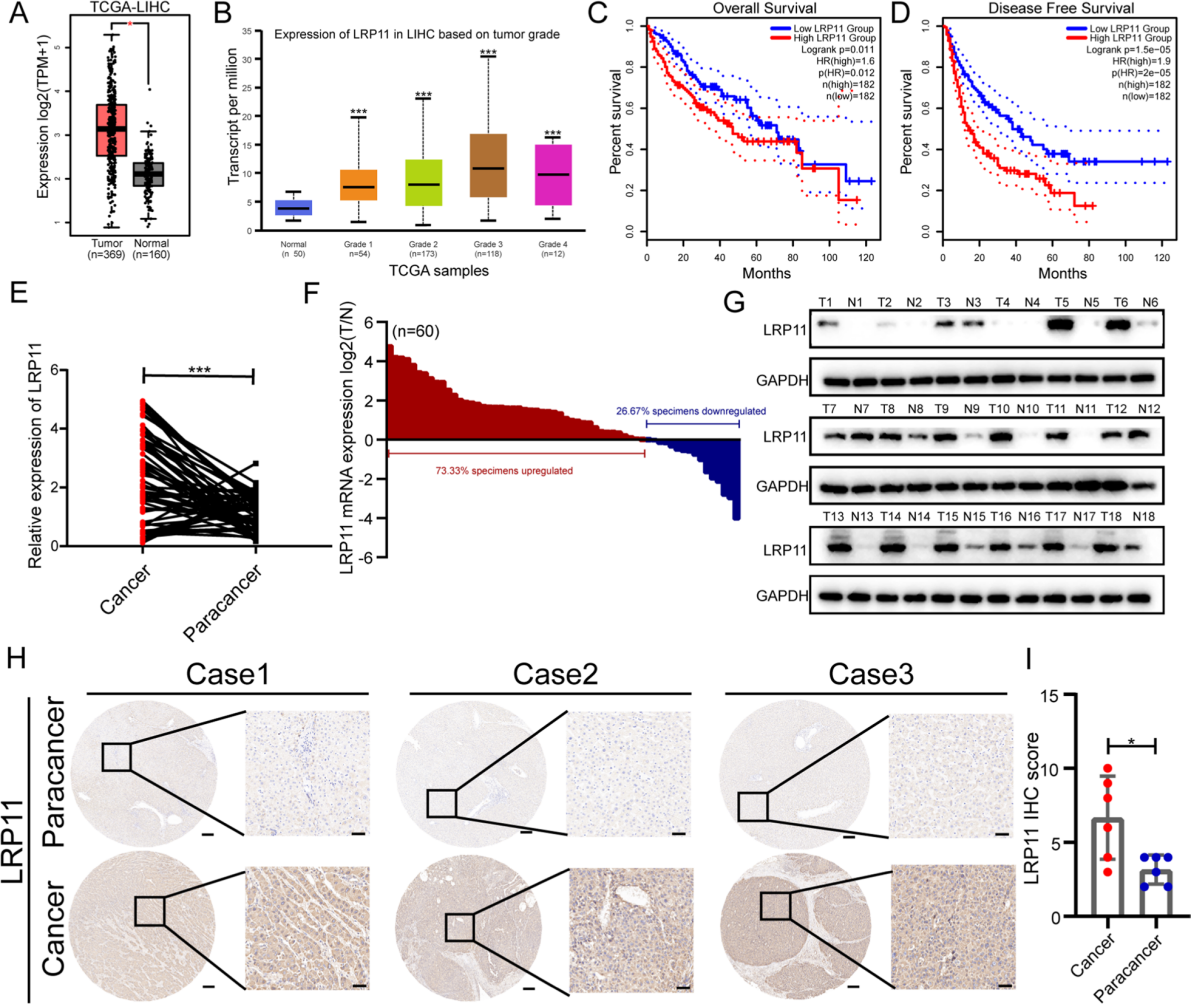
LRP11 upregulation is associated with HCC progression and poor prognosis. **A** The expression of LRP11 in TCGA-LIHC dataset. **B** Expression of LRP11 in LIHC on tumor grade. **C** The overall survival of patient in LRP11-low or LRP11-high expression group. **D** The disease-free survival of LIHC. n = 264. **E–F** RT-qPCR analysis of LRP11 mRNA expression in HCC tissues and paracancerous tissues. **G** Western blot assay was conducted to explore the protein levels of LRP11 in HCC tissues and matched normal tissues. **H-I** Immunohistochemical staining of LRP11 protein expression in human HCC tissues (Case1:T1N0M0, Case2 and Case3: T2N0M0) and their corresponding paracancerous tissue samples. Scale bar, 50 μm. All data are expressed as the mean ± SD of values from experiments performed in triplicate. *P < 0.05, **P < 0.01, ***P < 0.001

To further investigate key pathogenic factors in HCC, we analyzed the GSE14520 dataset and GEPIA database, LRP11 expression was found to be elevated in HCC tissues compared to non-tumor tissues. Analysis from GEPIA (Fig. 1A) and UALCAN (Fig. 1B) showed that higher LRP11 levels correlated with increased histological grade and reduced overall and disease-free survival (n = 364) (Fig. 1C, D). This suggests that LRP11 may act as an oncogene in HCC. Meanwhile, related studies have shown that LRP11 is involved in the malignant progression of various tumors, but its role in hepatocellular carcinoma remains unclear. Therefore, we first assessed the expression of LRP11 in HCC tissues. RT-qPCR revealed significantly higher LRP11 mRNA levels in 60 HCC samples compared to paired non-tumor tissues (Fig. 1E-F). Western blot analysis of 18 paired samples confirmed LRP11 protein upregulation in HCC tissues (Fig. 1G), demonstrating the consistency between mRNA and protein expression. Immunohistochemistry in six pairs of HCC and non-tumor tissues further supported these results (Fig. 1H–I). Additionally, LRP11 expression was positively correlated with tumor size and stage (Table 1). These findings indicate that LRP11 may be a potential marker of HCC progression and poor prognosis, warranting further investigation as a possible prognostic biomarker or therapeutic target.

**Table 1:** Correlation between LRP11 score in HCC and clinicopathological factors

Clinicopathological factors	No. of patients	LRP11		P value
	n = 60	High	Low	
Gender				
Male	39	19	20	0.787
Female	21	11	10	
Age (years)				
< 60	41	22	19	0.405
≥ 60	19	8	11	
AFP				
≤ 20	22	14	8	0.107
> 20	16	16	22	
HBsAg				
Negative	16	7	9	0.559
Positive	44	23	21	
Tumor number				
Single	48	22	26	0.333
Multiple	12	8	4	
Tumor size in cm				
≤ 5	28	10	18	0.038*
> 5	32	20	12	
TNM stage				
I	35	13	22	0.018*
II–III	25	17	8	
Microvascular invasion				
Yes	19	8	11	0.405
No	41	22	19	

*AFP* alpha-fetoprotein, *HBsAg* hepatitis B surface antigen

*p < 0.05; **p < 0.01

### *LRP11 promotes malignant progression of HCC *in vitro

**Fig. 2:**
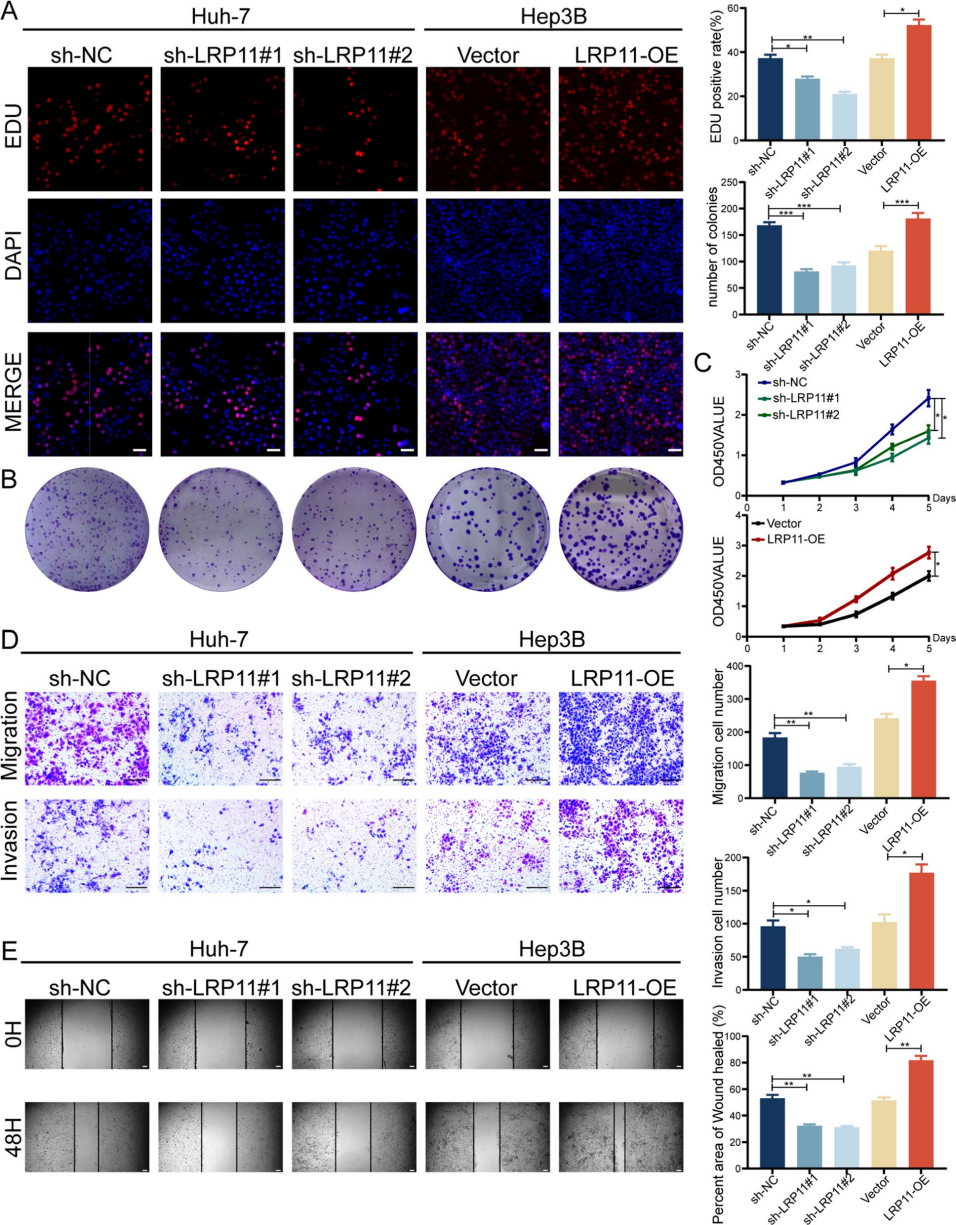
LRP11 promotes malignant progression of HCC in vitro.** A** Cell proliferation in HCC cells post-transfection with sh-NC, sh-LRP11#1, sh-LRP11#2, Vector, or LRP11-OE was evaluated using an EdU assay. Scale bar, 50 μm. **B** Cell proliferation was detected by knocking down LRP11 or overexpression LRP11 using colony formation assays. **C** Using CCK-8 assays detected the HCC cell's proliferation ability.** D** The transwell assay of HCC cells transfected with different plasmids. Scale bar, 500 μm. **E** Wound healing assay assessed the migratory capacity of Huh7 and Hep3B cells post-transfection with different plasmids. Scale bar, 100 μm. All data are expressed as the mean ± SD of values from experiments performed in triplicate. *P < 0.05, **P < 0.01, ***P < 0.001

To assess the role of LRP11 in HCC, we extracted protein and mRNA from the normal hepatocyte cell line HHL-5 and the HCC cell lines (SK-Hep1, Hep3B, Huh7, YY-8103, MHCCLM3, HepG2, MHCC97L) for comparison. LRP11 expression levels were then analyzed using Western blot and RT-qPCR (Fig. S1A). LRP11 expression was significantly higher in HCC cell lines compared to the normal hepatocytes, with the highest expression observed in Huh7 cells and the lowest in Hep3B cells. LRP11 overexpression was induced in Hep3B cells, while LRP11 knockdown (sh-LRP11#1, sh-LRP11#2) was achieved in Huh7 cells. Overexpression and knockdown efficiency were confirmed at both mRNA and protein levels (Fig. S1B-C). EdU (Fig. 2A), colony formation (Fig. 2B), and CCK-8 assays (Fig. 2C) demonstrated that LRP11 knockdown significantly inhibited cell proliferation, while its overexpression enhanced it. Transwell assays (Fig. 2D) and Wound healing assays (Fig. 2E) revealed that LRP11 knockdown suppressed, while overexpression promoted, cell migration and invasion. These results suggest that LRP11 may play a role in the malignant progression of HCC.

### LRP11 promotes lipid metabolism in HCC cells

**Fig. 3:**
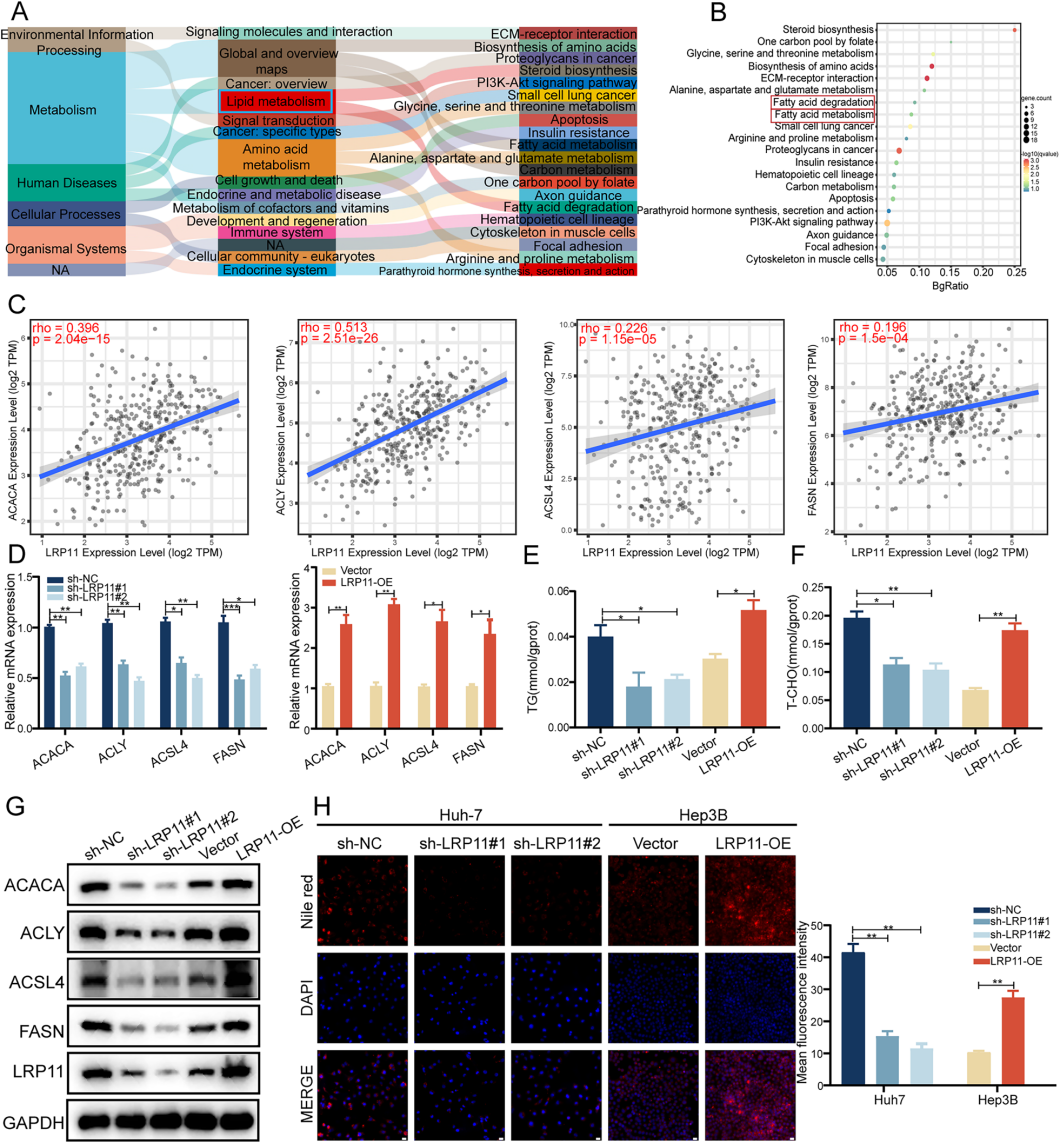
LRP11 promotes lipid metabolism in HCC cells.** A** Sankey diagram showing the trajectories of metabolite data flow across multiple pathways. **B** KEGG pathway enrichment analysis of the upregulated genes. **C** Scatter plot analysis of the correlation between LRP11 mRNA levels and ACACA, ACLY, ACSL4, and FASN in the Timer2.0 database. **D** RT-qPCR analysis of the mRNA expression of lipogenic enzyme genes ACACA, ACLY, ACSL4, and FASN in Huh7 and Hep3B cells. **E–F** Measurement of triglyceride and cholesterol levels in Huh7 and Hep3B cells.** G** Determination of lipogenic enzyme protein levels in Huh7 and Hep3B cells by Western blotting. **H** Measurement of intracellular neutral lipids in Huh7 and Hep3B cells using Nile red and DAPI double staining. Scale bar, 50 μm. This was followed by quantifying the mean fluorescence intensity of Nile red staining for each cell line. All data are expressed as the mean ± SD of values from experiments performed in triplicate. *P < 0.05, **P < 0.01, ***P < 0.001

To investigate the mechanism by which LRP11 promotes HCC progression, RNA-seq in LRP11-overexpressing Hep3B cells identified 1,234 upregulated genes compared to controls (Fig. S1D). Sankey diagram analysis highlighted metabolic pathways, particularly lipid metabolism, as the most affected by LRP11 overexpression (Fig. 3A). Complementing these findings, KEGG analysis further implicated fatty acid metabolism and degradation as key pathways impacted by LRP11 (Fig. 3B). Pearson correlation analysis using Timer2.0 database showed significant positive correlations between LRP11 and key lipogenic enzymes, including ACACA (r = 0.396, p < 0.0001), ACLY (r = 0.513, p < 0.0001), ACSL4 (r = 0.226, p < 0.0001), and FASN (r = 0.196, p < 0.0001) (Fig. 3C), all of which play crucial roles in lipid metabolism and fatty acid synthesis. RT-qPCR (Fig. 3D) and Western blot (Fig. 3G) confirmed decreased lipogenic enzyme expression in LRP11-knockdown Huh7 cells and increased expression in LRP11-overexpressing Hep3B cells. LRP11 knockdown reduced triglyceride and cholesterol levels, while overexpression increased them (Fig. 3E, F). Nile red staining demonstrated reduced lipid accumulation in LRP11-knockdown cells, whereas overexpression enhanced lipid accumulation (Fig. 3H). Taken together, these results suggest that LRP11 may play a significant role in regulating lipid metabolism and fatty acid synthesis in HCC cells, potentially contributing to the malignant progression of HCC.

### MAZ upregulates LRP11 in HCC

**Fig. 4:**
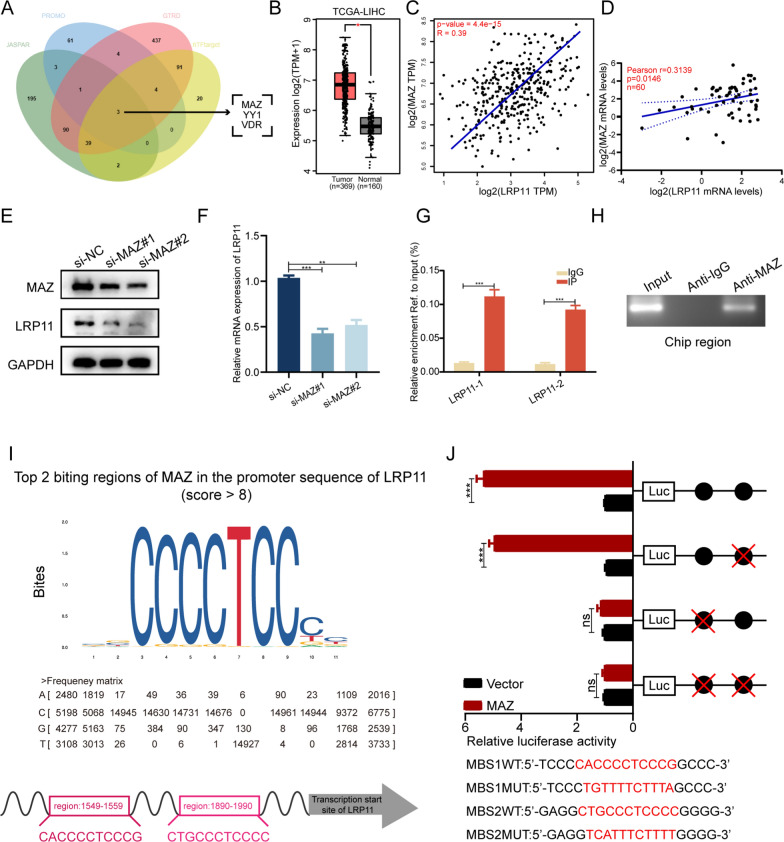
MAZ upregulates LRP11 in HCC.** A** Bioinformatics analysis identified MAZ, YY1, VDR as potential transcription factors driving LRP11 expression. **B** mRNA expression of MAZ in HCC from the GEPIA database. **C** Pearson correlation analysis shows a positive correlation between LRP11 and MAZ mRNA in the TCGA dataset. **D** Pearson correlation analysis shows a positive correlation between LRP11 and MAZ mRNA in the HCC tissue samples. **E, F** After MAZ knockdown, LRP11 mRNA and protein levels were reduced. **G-H** Chromatin immunoprecipitation (ChIP) combined with qRT-PCR and PCR provided evidence of MAZ binding to the promoter region of the LRP11 gene.** I** Putative MAZ binding sites (MBS1 and MBS2) were identified in the promoter region of the LRP11 gene. **J** Dual-luciferase reporter assays showed that mutation of MBS1 disrupted its ability to regulate the LRP11 promoter region. A red "X" within the binding region indicates the altered MAZ binding sequence. All data are expressed as the mean ± SD of values from experiments performed in triplicate. *P < 0.05, **P < 0.01, ***P < 0.001

To investigate the mechanism behind LRP11 overexpression in HCC, we used JASPAR, PROMO, GTRD, and hTFtarget databases to identify potential transcription factors that could activate LRP11 (Fig. 4A). MAZ, YY1, and VDR were identified as candidates. GEPIA database analysis identified that among the candidate transcription factors, MAZ expression is significantly elevated in HCC compared to non-tumorous tissues (Fig. 4B), while VDR and YY1 showed no significant differences in expression (Fig. S2A-B). Additionally, MAZ(Fig. 4C), VDR(Fig. S2C), and YY1(Fig. S2D) expression levels positively correlate with LRP11 mRNA expression. In addition, a positive correlation between MAZ and LRP11 mRNA expression was validated in 60 HCC tissue samples from our cohort (Fig. 4D). Western blot analysis demonstrated that MAZ knockdown resulted in a decreased LRP11 protein level (Fig. 4E). Consistently, RT-qPCR assays confirmed that silencing MAZ significantly reduced LRP11 expression in HCC cells (Fig. 4F), whereas knockdown of YY1 and VDR had no discernible effect on LRP11 mRNA levels (Fig. S2E-F), highlighting MAZ as the key regulator of LRP11 expression. These results suggest that MAZ may act as a potential transcriptional regulator of LRP11 in HCC. ChIP assays provided evidence of MAZ binding to the LRP11 promoter (Fig. 4G, H), and JASPAR predicted two MAZ binding sites (MBS1 and MBS2) (Fig. 4I). Dual-luciferase reporter assays with site-directed mutations in the LRP11 promoter showed that mutation of MBS1 abolished MAZ-induced LRP11 transcription (Fig. 4J). These findings support the hypothesis that MAZ could influence LRP11 transcription through promoter binding, potentially contributing to LRP11 overexpression in HCC.

### *LRP11 promotes HCC growth, metastasis, and lipid synthesis *in vivo

**Fig. 5:**
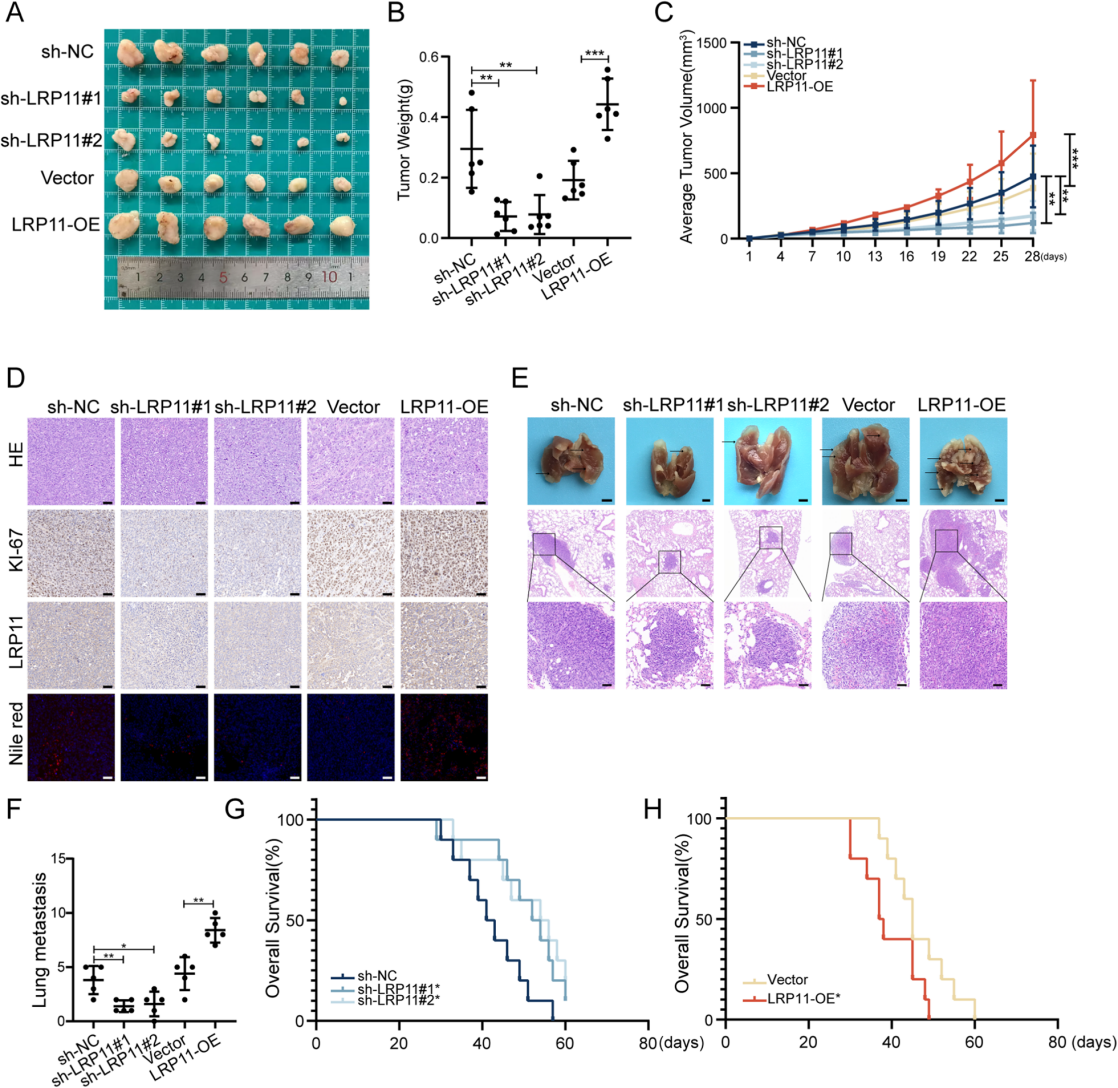
LRP11 promotes HCC growth, metastasis, and lipid synthesis in vivo**.** **A** Images of subcutaneous xenograft tumors from Huh7 (sh-NC, sh-LRP11#1, sh-LRP11#2) and Hep3B (Vector, LRP11-OE) cells. **B** Weight of the subcutaneous tumors. **C** Subcutaneous tumor volume growth curve. **D** H&E, Ki67, LRP11 IHC staining, and Nile red staining in xenograft tumors. Scale bar, 50 μm. **E** Images of lung metastases from Huh7 and Hep3B cells. Scale bar, 2 mm. Along with H&E staining of the metastatic lesions. Scale bar, 50 μm. **F** Quantification of lung metastases. **G, H** Kaplan–Meier survival curves of mice (n = 10). All data are expressed as the mean ± SD of values from experiments performed in triplicate. *P < 0.05, **P < 0.01, ***P < 0.001

To evaluate the effects of LRP11 on HCC in vivo, we established subcutaneous xenograft and lung metastasis models using Huh7 and Hep3B cells stably transfected with either LRP11 knockdown (sh-LRP11#1, sh-LRP11#2), overexpression (LRP11-OE), or control constructs (Fig. 5A). In the xenograft model, tumor size, weight, and proliferation rate were significantly reduced in the sh-LRP11 group compared to controls, while LRP11 overexpression had the opposite effect (Fig. 5B, C). IHC and Nile red staining showed reduced LRP11, KI-67, and lipid accumulation with LRP11 knockdown, and increased levels with LRP11 overexpression (Fig. 5D). In the lung metastasis model, LRP11 knockdown (sh-LRP11) significantly reduced both the size and number of metastatic nodules, as confirmed by H&E staining, while LRP11 overexpression (LRP11-OE) led to an increase in both metastatic burden and nodule size (Fig. 5E, F). Survival analysis revealed that mice injected with sh-LRP11 cells had prolonged overall survival (OS), while LRP11-OE mice had worse outcomes (Fig. 5G, H). These findings suggest that LRP11 promotes HCC growth, metastasis, and lipid synthesis in vivo, suggesting that LRP11 may serve as a potential therapeutic target for limiting tumor progression and metastasis in HCC.

### LRP11 interacts with RACK1

**Fig. 6:**
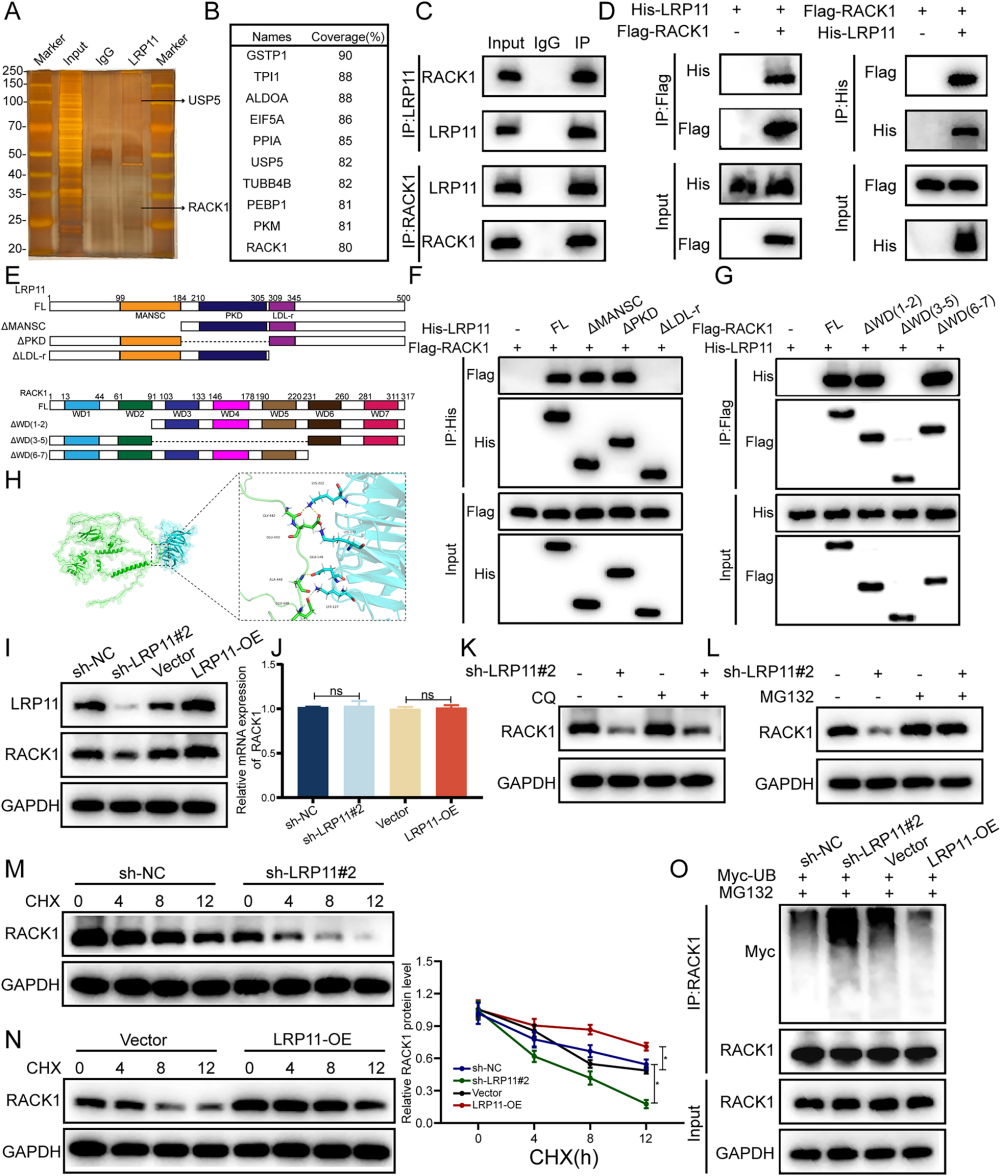
LRP11 mediates RACK1 function at the post-translational level. **A** Identification of interacting proteins using sensitive silver staining. **B** Mass spectrometry revealed LRP11 -interacting proteins. **C** Co-IP assays were performed to investigate the interaction between endogenous LRP11 and RACK1.** D** Co-IP assays were conducted in HEK293T cells to investigate the interaction between exogenous LRP11 and RACK1.** E** Schematic diagram of LRP11 domains and its truncated mutants (top) and RACK1 domains and its truncated mutants (bottom). **F–G** Co-IP assays were performed to investigate the binding regions between LRP11 and RACK1. **H** Schematic diagram of the binding sites between LRP11 and RACK1. **I, J** RACK1 mRNA and protein expression levels following LRP11 knockdown or overexpression. **K-L** RACK1 protein expression levels following LRP11 knockdown and treatment with CQ (10 µM,20 h) or MG132(10 µM,8 h). **M**–**N** RACK1 protein expression levels in Huh7 or Hep3B cells after CHX (50 μg/ml)treatment for the indicated times. **O** Ubiquitination of RACK1 following LRP11 knockdown or overexpression. All data are expressed as the mean ± SD of values from experiments performed in triplicate. *P < 0.05, **P < 0.01, ***P < 0.001. n.s. = not significant

To further explore the mechanism by which LRP11 regulates HCC progression, immunoprecipitation-mass spectrometry (IP-MS) and silver staining were performed to identify LRP11-interacting proteins (Fig. 6A, B). RACK1 was identified as a potential binding partner. This interaction was subsequently confirmed through exogenous and endogenous co-immunoprecipitation (Co-IP) assays (Fig. 6C, D). Molecular localization analysis revealed that residues 309–500 of LRP11 specifically interact with the RACK1 (residues 91–231) (Fig. 6E, G). These findings are further supported by docking simulation results, which provide additional evidence for the structural basis of the LRP11-RACK1 interaction (Fig. 6H).

### LRP11 mediates RACK1 function at the post-translational level

To elucidate the mechanism by which LRP11 and RACK1 influence HCC progression, Western blot (Fig. 6I) and RT-qPCR (Fig. 6J) were performed in HCC cells with LRP11 knockdown or overexpression. LRP11 knockdown inhibited RACK1 protein expression without affecting RACK1 mRNA levels, while LRP11 overexpression increased RACK1 protein expression, indicating that LRP11 regulates RACK1 at the post-translational level. In the presence of the protein synthesis inhibitor cycloheximide, RACK1 protein stability was enhanced in LRP11-overexpressing cells and reduced in LRP11-knockdown cells (Fig. 6M, N). Furthermore, treatment with the proteasome inhibitor MG132, but not the lysosomal inhibitor chloroquine, reversed the reduction of RACK1 protein levels caused by LRP11 knockdown (Fig. 6K, L), indicating that LRP11 may regulate RACK1 stability through the proteasomal degradation pathway. Moreover, LRP11 downregulation increased RACK1 ubiquitination, while LRP11 overexpression appeared to reduce it (Fig. 6O).

### LRP11 recruits USP5 to mediate RACK1 deubiquitination

**Fig. 7:**
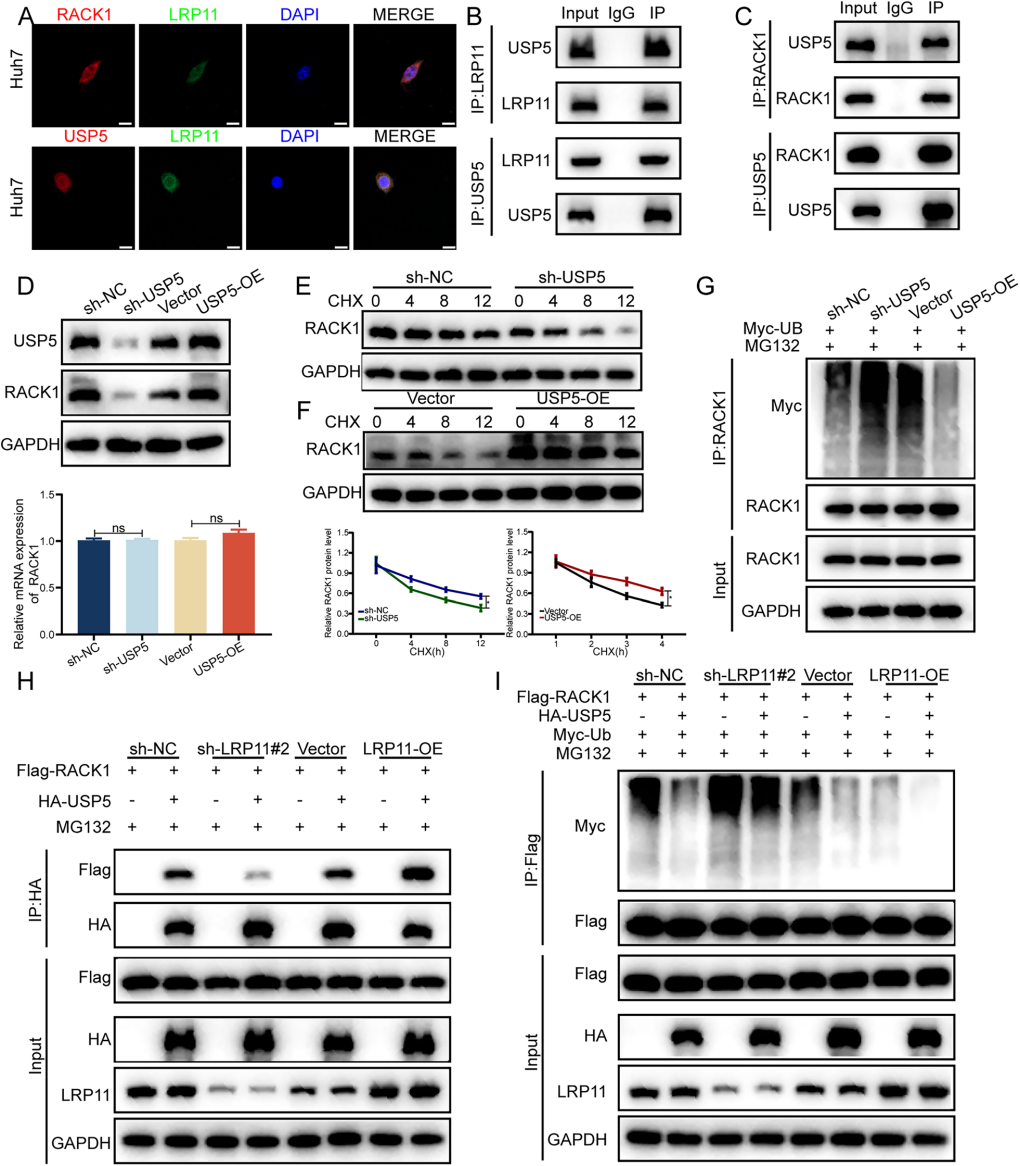
LRP11 recruits USP5 to mediate RACK1 deubiquitination.** A** Immunofluorescence of LRP11 (green), USP5(red)and RACK1 (red) in Huh7 cells. Scale bar, 20 μm. **B** Co-IP assays were performed to investigate the interaction between endogenous LRP11 and USP5.** C** Co-IP assays were performed to investigate the interaction between endogenous USP5 and RACK1. **D** RACK1 mRNA and protein expression levels following USP5 knockdown or overexpression. **E, F** RACK1 protein expression levels detected by Western blot in HCC cells treated with CHX and different plasmids. **G** Ubiquitination levels of RACK1 measured by Western blot after transfection with different plasmids. **H** Interaction between RACK1 and USP5 in LRP11 knockdown and overexpression cells. **I** Ubiquitination levels of RACK1 in LRP11 knockdown or overexpression cells with or without USP5. All data are expressed as the mean ± SD of values from experiments performed in triplicate. *P < 0.05, **P < 0.01, ***P < 0.001. n.s. = not significant

The results above indicate that LRP11 promotes RACK1 deubiquitination in HCC cells, despite not being a member of the deubiquitinase family. Thus, we hypothesized that LRP11 may regulate the interaction between RACK1 and its deubiquitinase. Based on this hypothesis, we identified USP5 as a candidate deubiquitinase for RACK1 through IP/MS screening. Immunofluorescence experiments demonstrated that LRP11 colocalizes and interacts with both RACK1 and USP5 in HCC cells (Fig. 7A, Fig. S2G). These interactions were further confirmed by exogenous and endogenous Co-IP assays (Fig. 7B, C, Fig. S2H-I). While our results confirmed the interaction between RACK1 and USP5, it was still uncertain whether USP5 directly mediates RACK1 deubiquitination, prompting further investigation. RT-qPCR and Western blotting revealed that USP5 knockdown decreased RACK1 protein levels without affecting RACK1 mRNA levels, confirming that USP5 regulates RACK1 at the post-translational level (Fig. 7D). Upon CHX treatment, RACK1 protein stability increased in USP5-overexpressing cells (Fig. 7E, F), and USP5 overexpression suppressed RACK1 ubiquitination (Fig. 7G). Importantly, When LRP11 is downregulated, the binding ability between USP5 and RACK1 is reduced. However, when LRP11 is overexpressed, the binding ability between USP5 and RACK1 is enhanced. LRP11 specifically promotes the interaction between USP5 and RACK1 (Fig. 7H). The ubiquitination experiments showed that the expression of USP5 reversed the increase in RACK1 ubiquitination induced by LRP11 knockdown, while also promoting the deubiquitination of RACK1 induced by LRP11 overexpression (Fig. 7I). In summary, we propose that LRP11 acts as a scaffold, facilitating the interaction between RACK1 and USP5, forming a regulatory complex that promotes RACK1 deubiquitination and stability. This regulatory axis may play a critical role in the progression of HCC.

### RACK1 and USP5 are critical for LRP11-mediated malignant phenotypes and metabolism in HCC

**Fig. 8:**
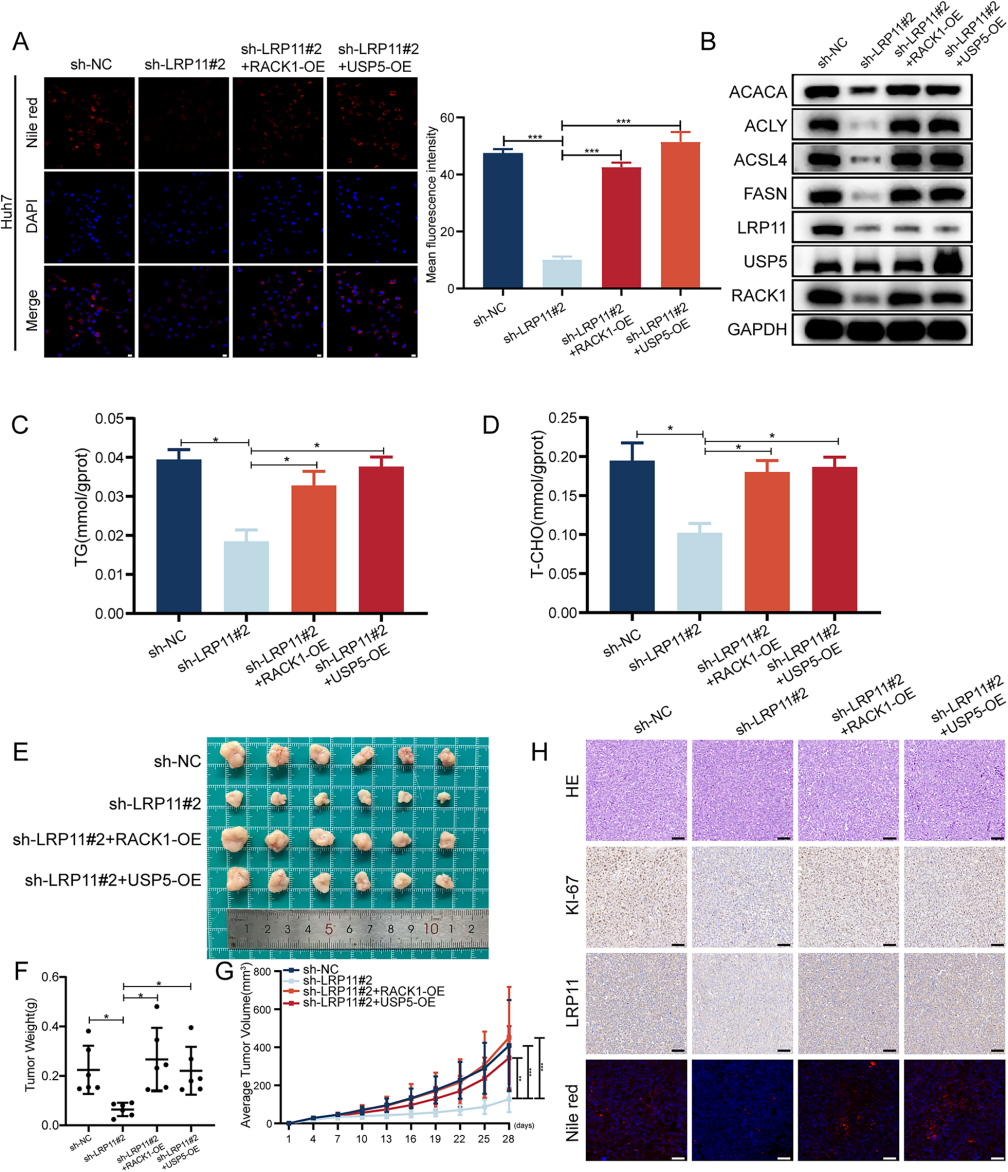
RACK1 and USP5 are critical for LRP11-mediated malignant phenotypes and metabolism in HCC.** A** Measurement of neutral lipids in Huh7 cells transfected with sh-NC, sh-LRP11#2, sh-LRP11#2 + RACK1-OE, and sh-LRP11#2 + USP5-OE plasmids using Nile red and DAPI double staining. Scale bar, 50 μm. This was followed by quantifying the mean fluorescence intensity of Nile red staining for each cell line. **B** Measurement of lipogenic enzyme protein levels in Huh7 cells transfected with different plasmids using Western blotting. **C**, **D** Measurement of triglyceride and cholesterol levels in Huh7 cells.** E** Images of subcutaneous xenograft tumors from Huh7 (sh-NC, sh-LRP11#2, sh-LRP11#2 + RACK1-OE, sh-LRP11#2 + USP5-OE) cells. **F** Weight of the subcutaneous tumors. **G** Subcutaneous tumor volume growth curve. **H** H&E, Ki67, LRP11 IHC staining, and Nile red staining in xenograft tumors. Scale bar, 50 μm. All data are expressed as the mean ± SD of values from experiments performed in triplicate. *P < 0.05, **P < 0.01, ***P < 0.001

To further validate the roles of RACK1 and USP5 in mediating LRP11-driven malignant phenotypes in HCC, we conducted rescue experiments to determine if overexpression of RACK1 or USP5 could reverse the effects of LRP11 knockdown. EdU (Fig. S3A) and colony formation assays (Fig. S3B) demonstrated that LRP11 knockdown impaired HCC cell proliferation. However, overexpression of RACK1 or USP5 effectively rescued this inhibitory effect, restoring cell proliferation to near-control levels. Similarly, Transwell (Fig. S3D) and wound healing assays (Fig. S3E) showed that LRP11 knockdown suppressed cell migration and invasion, which was reversed by RACK1 or USP5 overexpression. Furthermore, Nile red staining revealed that the LRP11 knockdown-induced reduction in lipid accumulation was rescued by RACK1 or USP5 overexpression (Fig. 8A), indicating their role in restoring lipid metabolism. Western blot analysis showed that RACK1 and USP5 overexpression restored the levels of key lipid metabolism proteins that had been reduced by LRP11 knockdown (Fig. 8B). This rescue effect was further confirmed by biochemical measurements of triglyceride and cholesterol levels (Fig. 8C, D). In vivo experiments further demonstrated that overexpression of RACK1 and USP5 alleviated the inhibitory effects of LRP11 knockdown on tumor proliferation, confirming their crucial role in mediating LRP11-driven HCC growth (Fig. 8E–H). In summary, our rescue experiments suggest that LRP11 may contribute to HCC malignant progression through its interactions with USP5 and RACK1, identifying these proteins as potential mediators of LRP11-associated oncogenic processes and as possible therapeutic targets for further investigation in HCC.

## Discussion

HCC, as one of the most common and highly malignant tumors worldwide, is associated with high incidence and mortality (Sung et al. 2021). Therefore, understanding the mechanisms driving the malignant progression of HCC is of paramount importance. This study provides novel insights into the potential oncogenic role of LRP11 in HCC and suggests that the interaction between LRP11 and RACK1 may contribute to HCC progression.

In this study, we found that higher LRP11 expression correlates with poorer clinical outcomes, indicating a potential tumor-promoting role for LRP11 in HCC. Analysis of LRP11 expression in HCC cells and tumor tissues revealed elevated levels compared to normal tissues. Although lower expression of LRP11 was observed in some HCC tissues, this may be influenced by factors such as tumor heterogeneity and changes in the tumor microenvironment. Therefore, the low expression of LRP11 in certain tumor tissues does not necessarily indicate a complete loss of its oncogenic function, but may reflect adaptive changes in tumor cells in response to the microenvironment or growth conditions. Functional assays in vitro and in vivo demonstrated that LRP11 overexpression significantly promotes HCC cell proliferation, migration, and invasion, while LRP11 knockdown had the opposite effects. Additionally, in both xenograft and lung metastasis models, LRP11 overexpression markedly enhanced tumor growth, invasion, and metastasis, providing additional support for its potential oncogenic role in HCC.

**Fig. 9:**
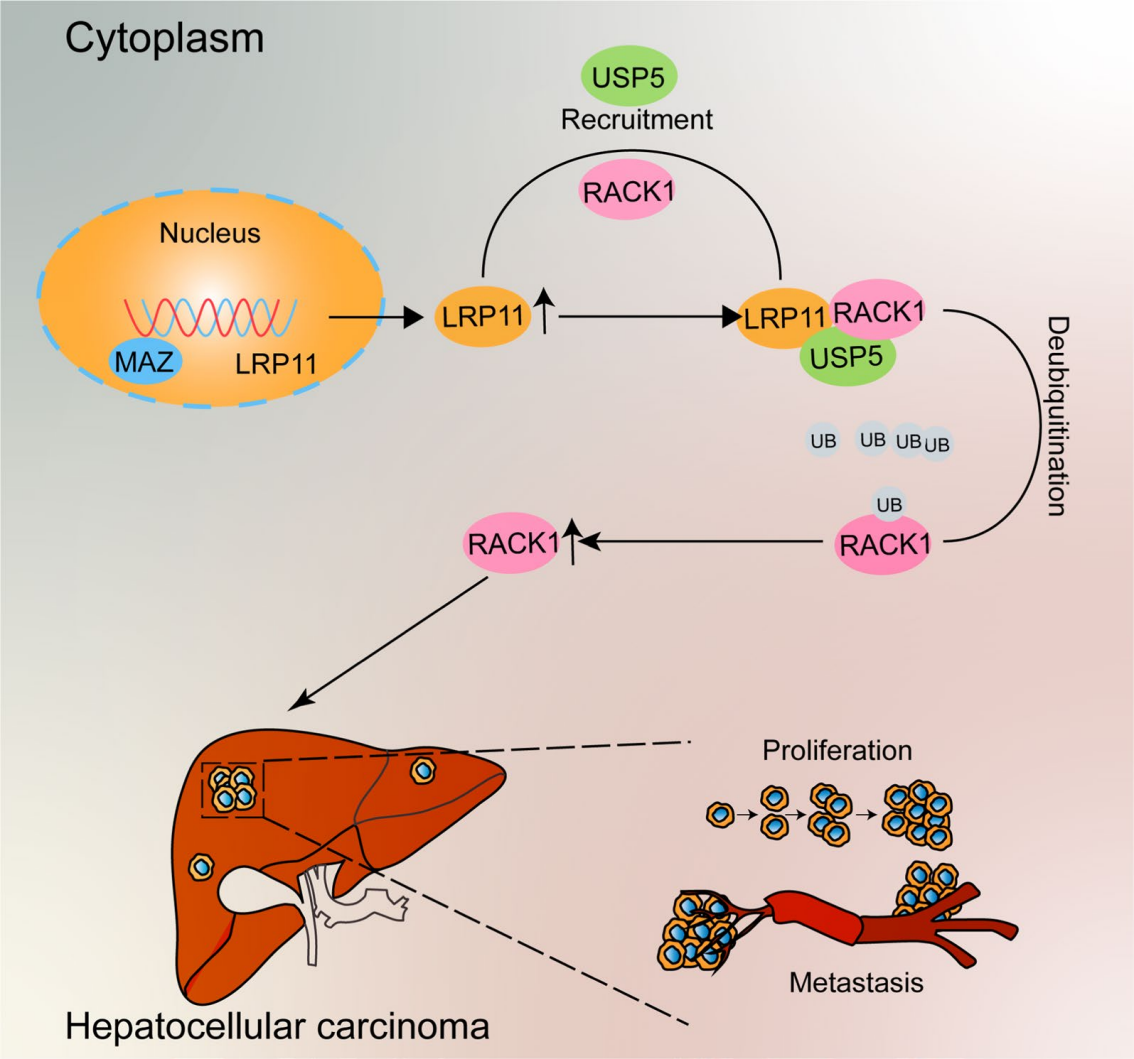
Diagram of the molecular mechanism

Lipid metabolism is a crucial component of tumor metabolism. Unlike normal human cells that rely on exogenous fatty acids, cancer cells preferentially synthesize lipids de novo to support their rapid proliferation (Medes et al. 1953). Studies have shown that aberrant lipid metabolism is a key feature of HCC. Elevated expression of genes associated with fatty acid (FA) biosynthesis in HCC tissues suggests that lipid metabolism plays a role in the pathogenesis of HCC (Berndt et al. 2019). FASN, ACACA, ACLY, and ACSL4 are considered key enzymes in lipid metabolism, and previous studies have shown that they can promote the proliferation of various cancers (Wei et al. 2023; Wang et al. 2010; Khwairakpam et al. 2015; Menendez and Lupu 2007; Quan et al. 2021). However, the mechanisms by which lipid metabolism functions in HCC cells remain unclear. A better understanding of fatty acid synthesis in HCC could help develop new therapeutic strategies for this malignancy. Through RNA sequencing, we found that LRP11 regulates a specific set of genes related to FA metabolism. The increase in fatty acid levels may provide energy for the uncontrolled proliferation of HCC cells (Fig. 9).

Mechanistic studies further demonstrated that LRP11 may promote tumorigenesis by interacting with RACK1 and stabilizing its protein levels through deubiquitination. RACK1, a highly conserved WD-repeat protein, is known to be dysregulated in multiple cancers. Previous research has shown that RACK1 can act as either a tumor suppressor or an oncogene, depending on the cancer type (Deng et al. 2012; Xiao et al. 2018), Moreover, elevated RACK1 expression promotes the malignant progression of HCC (Duan et al. 2018). This is the first study to identify the interaction between LRP11 and RACK1, contributing to the maintenance of RACK1 protein stability. Subsequent studies revealed that the 309–500 amino acid region of LRP11 is responsible for binding to the homologous domain of RACK1 (residues 91–231). Reducing LRP11 increases RACK1 ubiquitination and accelerates its degradation. These findings provide new insights into the mechanism by which LRP11 may contribute to HCC progression.

Ubiquitination is a post-translational modification that affects protein stability, intracellular transport, and enzyme activity, playing a critical role in regulating cellular functions. USP5 interacts with PD-1, leading to its deubiquitination and stabilization, thereby modulating tumor immunotherapy (Xiao et al. 2023). USP5 deubiquitination activity interacts with and stabilizes SLUG, thereby regulating its abundance in epithelial-mesenchymal transition (EMT) in HCC (Meng et al. 2019). Our findings attribute the stability of RACK1 to the regulation mediated by LRP11 through USP5. However, the specific binding sites between USP5 and RACK1 remain unclear, and it is uncertain whether other factors are involved in the regulation of RACK1 by LRP11. Whether these findings can be translated into clinical applications, such as therapeutic targets or diagnostic biomarkers, requires further clinical research to verify their efficacy and safety. These questions warrant additional experimental investigation.

In conclusion, our study suggests that LRP11 may play a role in promoting lipid metabolism, proliferation, migration, and invasion in HCC. Mechanistically, LRP11 appears to directly bind to RACK1 and enhance its stability by promoting deubiquitination through the recruitment of USP5, thereby potentially facilitating HCC lipid metabolism and progression. These findings indicate that LRP11 could be explored as a therapeutic target for HCC, although further research is needed to fully validate its clinical potential.

## Supplementary Information

Additional file 1.

Additional file 2.

Additional file 3.

## Data Availability

The data used were obtained from the TCGA database and the UALCAN dataset. All data generated or analyzed during this study are contained in this paper or supplementary materials. Processed data are available from the corresponding author upon reasonable request. No datasets were generated or analysed during the current study.

## Abbreviations

LRP11: LDL receptor related protein 11
RACK1: Receptor for activated C kinase 1
USP5: Ubiquitin specific peptidase 5
TCGA: The Cancer Genome Atlas
PVDF: Polyvinylidene Difluoride
