# Supplementary material for: LRP11 facilitates lipid metabolism and malignancy in hepatocellular carcinoma by stabilizing RACK1 through USP5 regulation
Source: Mol Med. 2025 Jan 31;31:35. doi: 10.1186/s10020-025-01097-6 (PMC11786360; doi:10.1186/s10020-025-01097-6)
Supplement: Supplementary file 1 — Additional file 1. [file 10020_2025_1097_MOESM1_ESM.docx]

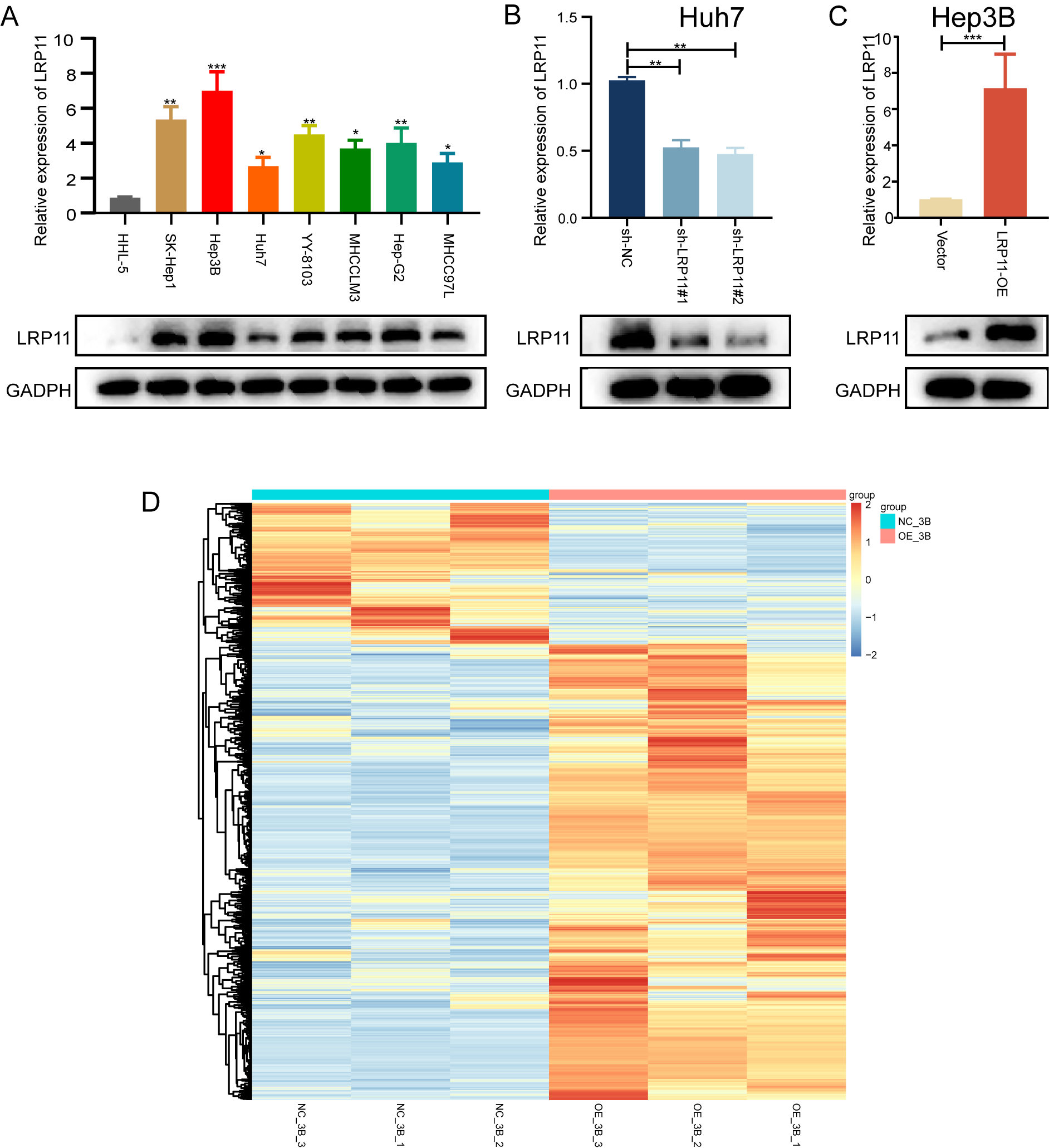


**Fig.S1 A** Western blot and RT-qPCR were used to detect the expression of LRP11 in HCC cell lines and liver normal cell lines, respectively. **B-C** Validation of LRP11 overexpression and knockdown efficacy by Western blot and RT-qPCR. **D** Heatmap showing gene expression changes following LRP11 overexpression in HCC cells. All data are expressed as the mean ± SD of values from experiments performed in triplicate. *P < 0.05, **P < 0.01, ***P < 0.001.


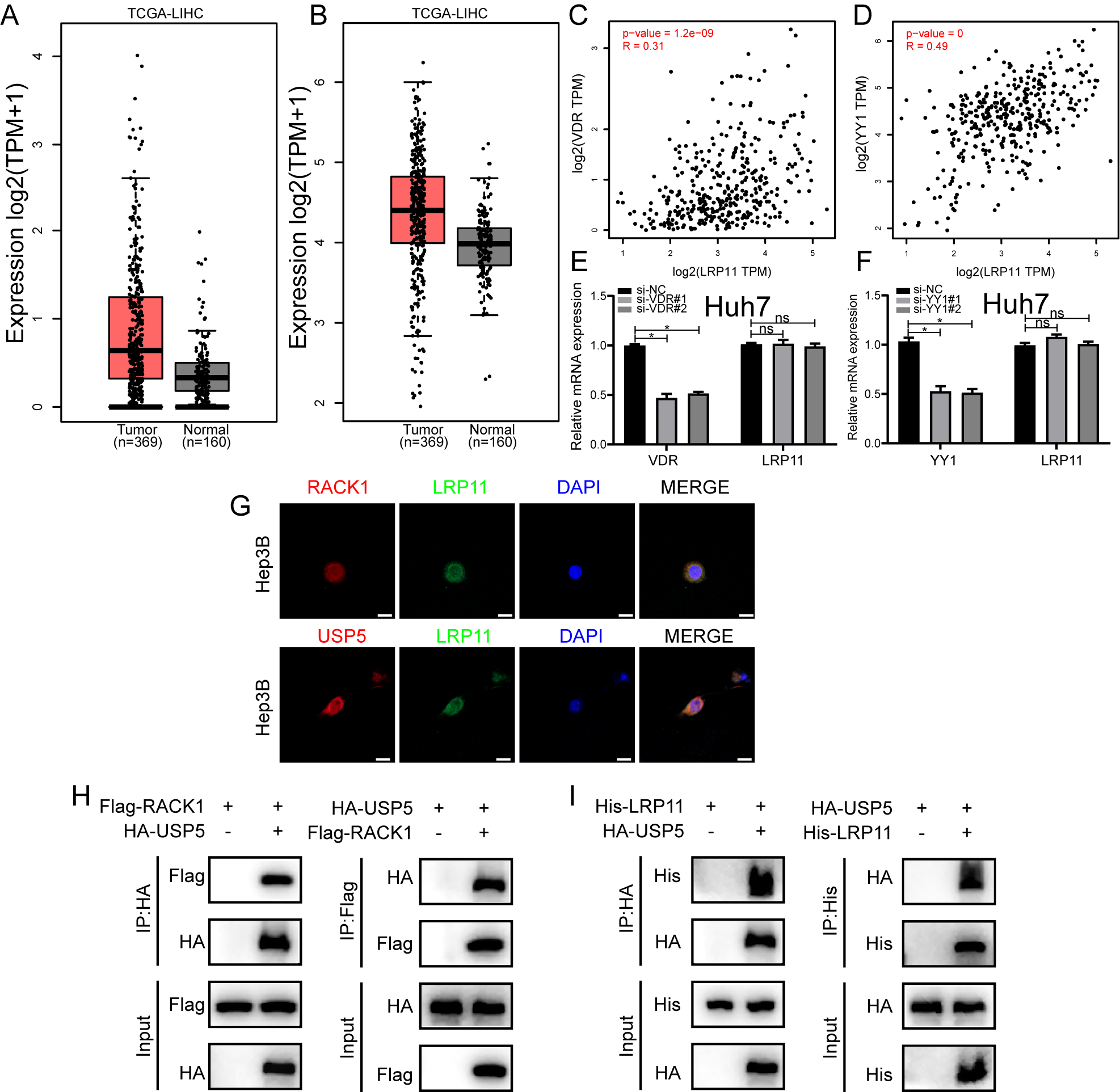


**Fig.S2 A-B** mRNA expression of VDR and YY1 in HCC from the GEPIA database. **C** Pearson correlation analysis shows a positive correlation between LRP11 and VDR mRNA in the TCGA dataset. **D** Pearson correlation analysis shows a positive correlation between LRP11 and YY1 mRNA in the TCGA dataset. **E** qRT-PCR analysis of LRP11 mRNA expression levels following VDR knockdown in the Huh7 cell line. **F** qRT-PCR analysis of LRP11 mRNA expression levels following YY1 knockdown in the Huh7 cell line. **G** Immunofluorescence of LRP11 (green), USP5(red)and RACK1 (red) in Hep3B cells. Scale bar, 20 μm. **H** Co-IP assays were conducted in HEK293T cells to investigate the interaction between exogenous USP5 and RACK1. **I** Co-IP assays were conducted in HEK293T cells to investigate the interaction between exogenous LRP11 and USP5. All data are expressed as the mean ± SD of values from experiments performed in triplicate. *P < 0.05, **P < 0.01, ***P < 0.001.


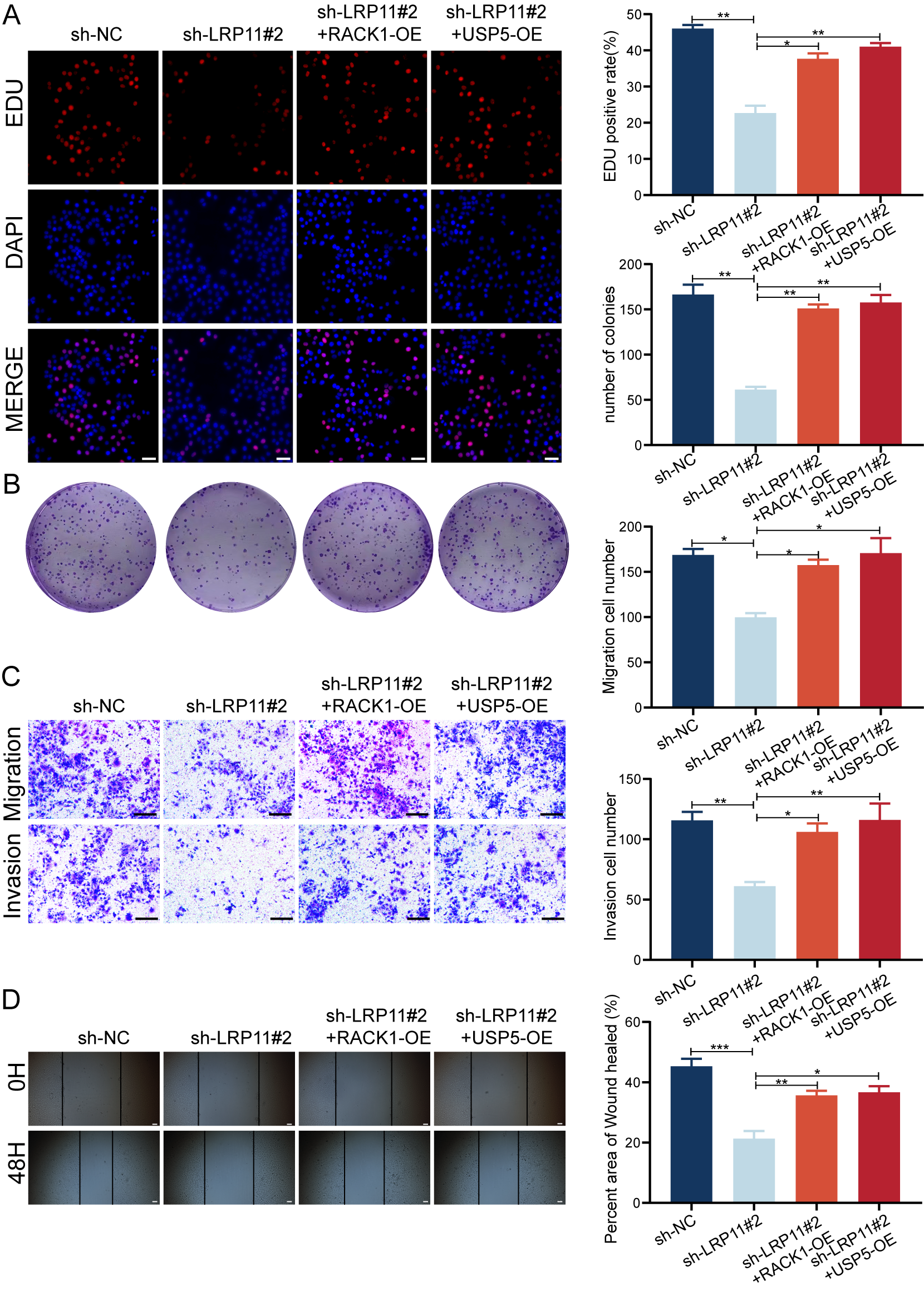


**Fig.S3 A** EdU assay to assess the proliferation of Huh7 cells transfected with the specified plasmids. Scale bar, 50 μm. **B** Colony formation assay to assess the proliferation of Huh7 cells transfected with the specified plasmids. Scale bar, 500 μm. **C** Transwell assay of Huh7 cells transfected with the specified plasmids. **D** Wound healing assay to assess the migration ability of Huh7 cells transfected with the corresponding plasmids. Scale bar, 100 μm. All data are expressed as the mean ± SD of values from experiments performed in triplicate. *P < 0.05, **P < 0.01, ***P < 0.001.
