# Supplementary material for: LRP11 facilitates lipid metabolism and malignancy in hepatocellular carcinoma by stabilizing RACK1 through USP5 regulation
Source: Mol Med. 2025 Jan 31;31:35. doi: 10.1186/s10020-025-01097-6 (PMC11786360; doi:10.1186/s10020-025-01097-6)
Supplement: Supplementary file 2 — Additional file 2. [file 10020_2025_1097_MOESM2_ESM.docx]

**Supplementary Table. S1 Primers for RT-qPCR analysis**

| **Gene** | **Primer Sequence (5'-3')** |
| --- | --- |
| **LRP11** | **Forward: GACCCGTCAGTGGACATGAA** |
|  | **Reverse: TCCCTCTTGGAACAGATGCAG** |
| **MAZ** | **Forward: ACTGTGGCAAGAGCTTCTCC** |
|  | **Reverse: TGGCACTTTCTCCTCGTGTC** |
| **FASN** | **Forward: AAGGACCTGTCTAGGTTTGATGC** |
|  | **Reverse: TGGCTTCATAGGTGACTTCCA** |
| **ACACA** | **Forward: ATGTCTGGCTTGCACCTAGTA** |
|  | **Reverse: CCCCAAAGCGAGTAACAAATTCT** |
| **ACLY** | **Forward: TCGGCCAAGGCAATTTCAGAG** |
|  | **Reverse: CGAGCATACTTGAACCGATTCT** |
| **ACSL4** | **Forward: CATCCCTGGAGCAGATACTCT** |
|  | **Reverse: TCACTTAGGATTTCCCTGGTCC** |
| **RACK1** | **Forward: TGAGTGTGGCCTTCTCCTCT** |
|  | **Reverse: TGATAGGGTTGCTGCTGTTG** |
| **USP5** | **Forward: GCTGCTGTCAGTATTACCGAC** |
|  | **Reverse: AAAGCCCAGAAACGTGTTCATA** |

**Supplementary Table. S2 Antibodies used in this study**

| **Antibody** | **Company** | **Product number** | **Dilution** | **Application** |
| --- | --- | --- | --- | --- |
| **LRP11** | **Santa Cruz** | **sc-514698** | **1:250** | **IF** |
|  |  |  | **1:1500** | **WB** |
| **MAZ** | **Abcam** | **Ab85725** | **1:1000** | **WB** |
| **FASN** | **Santa Cruz** | **sc-48357** | **1:1000** | **WB** |
| **ACACA** | **Santa Cruz** | **sc-137104** | **1:1000** | **WB** |
| **ACLY** | **Santa Cruz** | **sc-517267** | **1:1000** | **WB** |
| **ACSL4** | **Affinity** | **DF12141** | **1:1000** | **WB** |
| **UB** | **Cell Signaling Technology** | **58395S** | **1:1000** | **WB** |
| **FLAG** | **Proteintech** | **20543-1-AP** | **1:2000** | **WB** |
| **HIS** | **Proteintech** | **66005-1-Ig** | **1:1000** | **WB** |
| **MYC** | **Proteintech** | **10828-1-AP** | **1:2000** | **WB** |
| **HA** | **Proteintech** | **51064-2-AP** | **1:1000** | **WB** |
| **RACK1** | **Proteintech** | **27592-1-AP** | **1:1000** | **WB** |
|  |  |  | **1:250** | **IF** |
| **USP5** | **Proteintech** | **10473-1-AP** | **1:1000** | **WB** |
|  |  |  | **1:100** | **IF** |
| **IgG** | **Cell Signaling Technology** | **2729S** | **1:1000** | **WB** |
| **GADPH** | **Proteintech** | **60004-1-Ig** | **1:2000** | **WB** |

**Supplementary Table. S3 Sequence information of shRNAs, siRNAs**

| **Assay** | **Genes** | **Sequences** |
| --- | --- | --- |
| **sh-RNA** | **Sh-LRP11#1** | **5'-GATCCGAATCGGACTACCTCATAAATCTCG**  **AGATTTATGAGGTAGTCCGATTCTTTTTG-3'** |
| **sh-RNA** | **Sh-LRP11#2** | **5'-GATCCGAGTCAAATCATTCCTGTGATCTCG**  **AGATCACAGGAATGATTTGACTCTTTTTG-3'** |
| **si-RNA** | **Si-MAZ#1** | **5'-GGAAUUCGCUAGGUUUUAACGTT-3'** |
| **si-RNA** | **Si-MAZ#2** | **5'-GUUUUAACGAUUUGUUUCUCCTT-3'** |
| **sh-RNA** | **Sh-USP5** | **5'-CCGGGACCACACGATTTGCCTCATTCTCG**  **AGAATGAGGCAAATCGTGTGGTCTTTTTG-3'** |

**Supplementary Table 4: Primers sequences used for CHIP**

| **Gene** | **Primer Sequence (5'-3')** |
| --- | --- |
| **LRP11-1**  **Promoter1** | **Forward:** **GCGGGAGCAAAGTTGTTCTGA** |
|  | **Reverse: GGTGGAGCCTTGGTGAGC** |
| **LRP11-2**  **Promoter2** | **Forward: CCACCTCACCAGGCTTGATT** |
|  | **Reverse: AGATGTGCTTGGGTAGTACAAAGT** |
