# Supplementary material for: LRP11 facilitates lipid metabolism and malignancy in hepatocellular carcinoma by stabilizing RACK1 through USP5 regulation
Source: Mol Med. 2025 Jan 31;31:35. doi: 10.1186/s10020-025-01097-6 (PMC11786360; doi:10.1186/s10020-025-01097-6)
Supplement: Supplementary file 3 — Additional file 3. [file 10020_2025_1097_MOESM3_ESM.docx]

**Supplementary Materials and methods**

**Cell lines**

All cell lines were cultured in Dulbecco’s modified Eagle medium (DMEM; Gibco, Carlsbad, CA, USA) supplemented with 10% fetal bovine serum (Gibco), 50 U/mL penicillin (Invitrogen, Waltham, MA, USA), and 50 U/mL streptomycin (Invitrogen). All cells were maintained in a humidified incubator at 37°C with 5% CO₂. The cell lines used in this study include the HEK 293T cell line, the liver normal cell line HHL-5 and following human HCC cell lines Huh7, MHCC97L, SK-Hep1, YY 8103(from the Shanghai Bio-Cell Bank), and the cell lines HepG2, Hep3B, MHCCLM3 (from Shanghai Institute of Cell Biology, Chinese Academy of Sciences).

**Quantitative real-time polymerase chain reaction (qRT-PCR)**

Total RNA was extracted from cells or tissues using TRIzol reagent (Invitrogen), and RNA concentration was measured with a spectrophotometer. RNA was reverse-transcribed into cDNA using a reverse transcription kit (VA zyme, Nanjing, China). Quantitative PCR analysis of the resulting cDNA was performed using Ace qPCR SYBR Green Master Mix (VA zyme) and an ABI 7900 PCR system (Applied Biosystems Inc., Waltham, MA, USA). The relative mRNA levels were normalized to GAPDH and determined using the 2^-ΔΔCt^ method. Primer sequences are provided in Supplementary Table S1.

**Western blot**

Total protein was extracted from cells or tissues using RIPA buffer, separated by SDS-PAGE, and transferred onto PVDF membranes. The membranes were blocked with 5% skim milk for 2 hours and incubated overnight at 4 °C with primary antibodies. The PVDF membranes were then incubated with appropriate secondary antibodies at room temperature for 2 hours. Protein bands were detected using enhanced chemiluminescence (ECL, Bioshrap, China). Details of the primary antibodies are provided in Supplementary Table S2.

**Immunohistochemistry (IHC)**

For IHC staining, collected subcutaneous tumors, HCC, and adjacent normal tissues were fixed in formalin for 14 hours, embedded in paraffin, and sectioned into 5 μm thick slices. The sections were incubated with primary antibodies overnight at 4 °C. The secondary antibody was then applied using a 50 mM Tris-HCl (pH 7.6) solution containing 0.05% DAB and 0.03% H2O2, resulting in a brown-colored reaction. Finally, the slides were counterstained with hematoxylin, dehydrated, and mounted with resin for IHC analysis.

**Cell transfection and lentiviral infection**

In human HCC cells (Hep3B and Huh7), we constructed LRP11 plasmids and lentiviral packaging (GenePharma, China). Human shRNA (GenePharma, China) was used to suppress LRP11 expression in Huh7. Initially, 1 × 10^5 cells were seeded into six-well plates and incubated with 2 mL of culture medium for 24 hours. After this, 2 mL of complete medium was added along with the appropriate amount of virus and 40 μL of polybrene (Sigma-Aldrich, USA). The cells were incubated for 12-16 hours, then placed in normal culture medium and subjected to puromycin selection. Transfection efficiency was monitored by qRT-PCR and Western blotting. For overexpression experiments, LRP11 cDNA was inserted into the pcDNA3.1 vector. Lipofectamine 3000 (Invitrogen, USA) was used to facilitate transfection of the Hep3B with the plasmid, with the pcDNA3.1 vector serving as the control. The target sequences are listed in Supplementary Table S3.

**Colony formation and EdU assays**

Both Huh7 and Hep3B cells (800-1000 cells per well) were seeded into six-well plates and cultured for two weeks. Cells were then fixed with 4% paraformaldehyde and stained with crystal violet solution. For the EdU assay, HCC cells (50,000 cells per well) were seeded in triplicate into 24-well plates and incubated for 24 hours. The cells were incubated with 10 μM EdU solution at 37°C for 2 hours, followed by fixation with 4% paraformaldehyde. The experiment was performed according to the EdU kit (RIBOBIO, Guangzhou, China) instructions, and images were captured using a fluorescence microscope.

**Cell viability assay**

Both Huh7 and Hep3B cells were seeded into 96-well plates at a density of 2,000 cells per well, with 100 μL of cell suspension added to each well, and three replicates were set for each treatment group. Then, 10 μL of CCK-8 reagent (Biosharp, Beijing, China) was added to each well, and the cells were incubated for 2 hours at 37°C. The optical density (OD) at 450 nm was measured using a microplate reader. Cell proliferation was monitored for 5 consecutive days, and a proliferation curve was generated.

**Wound healing assay**

Both Huh7 and Hep3B cells (5 × 10^5 cells) were seeded into six-well plates. When the cells reached 90% confluence, a 200 μL pipette tip was used to scratch the cell surface. After 48 hours, the wound closure was calculated using the formula: wound closure = (wound width at 0 hours − wound width at 48 hours) / wound width at 0 hours × 100%. Images were captured using an inverted phase-contrast microscope.

**Transwell assay**

Uncoated transwell chambers (8 µm pore size; Corning, USA) were used for migration assays, while 50 µL of Matrigel (1:10; BD Biosciences, USA) was used to pre-coat the upper surface of the transwell chambers for invasion assays. 1 × 10^4 Huh7 or Hep3B cells (suspended in 200 µL serum-free medium) were seeded into the upper chamber, while 600 µL medium supplemented with 10% FBS was added to the lower chamber. After 48 hours of incubation, the cells on the lower surface of the membrane were fixed with 4% paraformaldehyde for 30 minutes and stained with crystal violet. Cells from three randomly selected fields were imaged and counted. The experiment was performed in triplicate.

**TG and THO measurements**

1 × 10^6 cells of Hep3B overexpressing LRP11, Huh7 cells with LRP11 knockdown, and corresponding control cells were collected. The cell samples were lysed with 100 μL of 2% Triton X-100 (Biosharp, China) lysis buffer. Total cholesterol and triglyceride levels were measured using the Total Cholesterol Assay Kit (Njjcbio, China) or Triglyceride Assay Kit (Njjcbio, China), with blank, calibration, and sample groups prepared. After adding 250 μL of working solution to each group, distilled water, calibrator, and cell samples were added to the blank, calibration, and sample groups, respectively, and incubated at 37°C for 10 minutes. Absorbance at 500 nm was measured using a microplate reader. Total cholesterol and triglyceride contents were calculated using the following formula: T-CHO/TG (mmol/gprot) = [(A_sample_ − A_blank_) / (A_calibration_ − A_blank_)] * C_calibration_ / Cpr (C_calibration_: concentration of the calibration; Cpr: concentration of the sample).

**Nile red staining**

The HCC cell lines (Huh7 and Hep3B) were counted and seeded into six-well plates at a density of 1 × 10^5 cells per well, 48 hours post-transfection. After 24 hours, the cells were fixed with 4% paraformaldehyde for 30 minutes. The cells were then stained using the Nile Red Staining Kit (Beyotime, Shanghai, China) according to the manufacturer's instructions, and incubated at room temperature for 20 minutes. Fluorescent images were captured using an inverted fluorescence microscope (Nikon, Japan). The experiment was performed in triplicate.

**ChIP assay**

Huh7 cells were crosslinked with 1% formaldehyde in PBS at 25°C for 10 minutes. The reaction was quenched by adding 0.125 M glycine, and cells were lysed on ice with lysis buffer. Chromatin DNA was sonicated and incubated overnight at 4°C with anti-MAZ antibody. The chromatin bound to MAZ was precipitated using protein A/G agarose beads (Thermo Scientific, MA, USA). After reversing the crosslinks, the purified DNA was used as a PCR template for analysis via qPCR and PCR to validate the binding of MAZ to the LRP11 promoter region. Primers used for this analysis are listed in Supplementary Table S4.

**Dual-luciferase reporter assay**

HEK293T cells were cultured in 24-well plates until 80% confluence. The cells were then transfected with mutant LRP11 promoter luciferase reporter plasmids and either MAZ expression plasmids or control vectors. After cell lysis, firefly luciferase activity was measured using a dual-luciferase reporter assay kit (Beyotime, Shanghai, China) and normalized to Renilla luciferase activity.

**Xenograft nude mouse model**

Four-week-old male nude mice (Vital River, Beijing, China) were divided into four groups, with six mice per group. The mice were maintained in a specific pathogen-free (SPF) environment. A total of 5 million lentivirus-transfected HCC cells were injected into the left axilla of each mouse. Subcutaneous tumor volumes were recorded every three days for 28 days, after which the mice were euthanized. The subcutaneous tumors were excised, photographed, and analyzed for immunohistochemistry and volume measurement. Tumor volume was calculated using the formula: volume = length × (width^2) / 2. All animal experiments in this study were approved by the Institutional Animal Care and Use Committee (IACUC) of the First Affiliated Hospital of Nanjing Medical University, and all procedures were conducted in accordance with IACUC guidelines.

**Lung metastasis model**

Four-week-old male BALB/c nude mice (Vital River, Beijing, China) were divided into four groups, with 16 mice per group. A total of 1.5 million experimental cells were injected via the tail vein. After four weeks, six randomly selected mice from each group were euthanized, and their lungs were collected for photography and hematoxylin and eosin (HE) staining. The remaining mice (10 per group) were monitored for survival analysis over a two-month observation period.

**Ubiquitination assay**

HCC and HEK 293T cells were transfected with the indicated plasmids with or without Myc-Ub for 48 hours, followed by treatment with 10 μM proteasome inhibitor MG132 for an additional 8 hours. Cells were then collected for CO-IP. Ubiquitinated RACK1 was detected by Western blot using an anti-Myc antibody.

**Immunofluorescence**

Coverslips were placed at the bottom of 24-well plates, and 40,000 treated HCC cells were added to each well. Once the cells adhered, they were fixed with 4% paraformaldehyde and blocked with goat serum for 30 minutes. The cells were incubated overnight at 4°C with the primary antibody. After three washes with PBST, the cells were incubated with fluorescent secondary antibodies (Beyotime, Shanghai, China) for 1 hour, followed by nuclear staining with DAPI (Beyotime, Shanghai, China). Images were captured using a confocal microscope.
